# Nivalenol Has a Greater Impact than Deoxynivalenol on Pig Jejunum Mucosa *in Vitro* on Explants and *in Vivo* on Intestinal Loops

**DOI:** 10.3390/toxins7061945

**Published:** 2015-05-29

**Authors:** Sophal Cheat, Juliana R. Gerez, Juliette Cognié, Imourana Alassane-Kpembi, Ana Paula F. L. Bracarense, Isabelle Raymond-Letron, Isabelle P. Oswald, Martine Kolf-Clauw

**Affiliations:** 1 Université de Toulouse, Institut National Polytechnique-Ecole Nationale Vétérinaire (INP-ENVT), Unité Mixte de Recherche UMR 1331 Toxalim, Research Center in Food Toxicology, 23 chemin des Capelles, F-31300 Toulouse, France; E-Mail: julianarubira@hotmail.com (J.R.G.); 2 INRA, UMR 1331 Toxalim, Research Center in Food Toxicology, 180 chemin de tournefeuille F-31027 Toulouse, France; E-Mails: imourana.alassane-kpembi@toulouse.inra.fr (I.A.K.); ioswald@toulouse.inra.fr (I.P.O.); 3 Faculty of Animal Science and Veterinary Medicine, Royal University of Agriculture, P.O. box 2696, Phnom Penh, Cambodia; 4 Laboratory of Animal Pathology, Universidade Estadual de Londrina, 86057-990 Londrina, Brazil; E-Mail: anapaula@uel.br; 5 Plate-forme CIRE Chirurgie et Imagerie pour la Recherche et l’Enseignement UMR 085 PRC, INRA, 37380 Nouzilly, France; E-Mail: juliette.cognie@tours.inra.fr; 6 Hôpital d’Instruction des Armées, Camp Guézo 01BP517 Cotonou, Benin; 7 INP-ENVT, Université de Toulouse, F-31300 Toulouse, France; 8 STROMALab UMR5273 UPS EFS INSERM U1031, 1 Avenue Jean Poulhes, 31403 Toulouse, France; E-Mail: i.raymond@envt.fr

**Keywords:** mycotoxins, jejunum explant, loops, deoxynivalenol, nivalenol, enterocytes, histomorphology

## Abstract

The mycotoxins deoxynivalenol (DON) and nivalenol (NIV), worldwide cereal contaminants, raise concerns for animal and human gut health, following contaminated food or feed ingestion. The impact of DON and NIV on intestinal mucosa was investigated after acute exposure, *in vitro* and *in vivo*. The histological changes induced by DON and NIV were analyzed after four-hour exposure on pig jejunum explants and loops, two alternative models. On explants, dose-dependent increases in the histological changes were induced by DON and NIV, with a two-fold increase in lesion severity at 10 µM NIV. On loops, NIV had a greater impact on the mucosa than DON. The overall proliferative cells showed 30% and 13% decrease after NIV and DON exposure, respectively, and NIV increased the proliferative index of crypt enterocytes. NIV also increased apoptosis at the top of villi and reduced by almost half the proliferative/apoptotic cell ratio. *Lamina propria* cells (mainly immune cells) were more sensitive than enterocytes (epithelial cells) to apoptosis induced by NIV. Our results demonstrate a greater impact of NIV than DON on the intestinal mucosa, both *in vitro* and *in vivo*, and highlight the need of a specific hazard characterization for NIV risk assessment.

## 1. Introduction

Fungi of the *Fusarium* genus commonly contaminate cereals in the temperate climatic zones of the world and contribute to poor quality grains entering the feed and food chain. Among the mycotoxins produced by *Fusarium*, the large group of trichothecenes is extremely prevalent, particularly deoxynivalenol (DON) for which many exposure and toxicological surveys have been carried out or reviewed recently [1,2]. Nivalenol (NIV), classified with DON as a type B trichothecene, is a biologically active metabolite of DON, present in agricultural commodities [3,4]. A large-scale data survey has indicated that DON and NIV are present in 57% and 16%, respectively, of food samples collected in the European Union [5].

From their first discovery, there has been concern about the relationship between trichothecenes exposure and health damage based on both experimental toxicity and epidemiological data. Studies have shown that mycotoxins cause toxic effects in humans as well as in all animal species so far investigated, the pig being the most sensitive species [6]. Studies in laboratory and farm animals have revealed a complex spectrum of toxic effects. Experimentally, low to moderate acute oral exposure to trichothecenes cause vomiting, diarrhea and gastroenteritis, whereas higher doses cause severe damage to the lymphoid and epithelial cells of the gastrointestinal mucosa resulting in hemorrhage, endotoxemia and shock. Chronic exposure to trichothecenes can cause anorexia, reduced weight gain, diminished nutritional efficiency, neuroendocrine changes, and immune modulation. Although not as prevalent as DON [7], NIV showed higher acute toxicity than DON in animal studies, with oral LD_50_ values in mice of 78 and 39 mg·kg^−1^ for DON and NIV, respectively [8]. NIV is of added concern for food safety but *in vivo* information for assessing the health risk remain scarce, and NIV toxicity is considered similar to DON toxicity for protecting human health [9]. At the molecular level, DON and NIV, like other trichothecenes, display multiple inhibitory effects on the primary metabolism of eukaryotic cells including the inhibition of proteins, DNA and RNA synthesis [10]. This impairment leads to altered cell proliferation in tissues with high rates of cell turnover such as spleen, bone marrow, thymus and intestinal mucosa [11].

Following ingestion of contaminated feed or food, the intestine and intestinal mucosa can be exposed to a high concentration of food contaminants, such as mycotoxins [12]. However, only a few studies have investigated the effects of mycotoxins on this target, even though there is increasing evidence that intestinal epithelium is repeatedly exposed to mycotoxins at a higher concentration than other tissues [13]. Pigs receiving 3 mg/kg feed of DON for five weeks showed significant histopathological changes compared to control animals, such as atrophy and fusion of villi and reduction of the number of goblet cells and lymphocytes [14]. Little is known about the effects of NIV on the intestinal tract of pigs. Pigs receiving 2.5 or 5 mg NIV/kg of feed for three weeks, showed gastrointestinal erosions [15] and reduced enzymatic ability of the intestinal epithelium [16].

In the context of implementing the 3Rs, “Replace, Reduce, Refine” [17], *in vitro* and *in vivo* alternatives can be used to reduce the number of experimental animals. An intestinal explants-*in vitro* model and an intestinal loops-*in vivo* model have been developed for studying intestinal responses to pathogens [18,19]. The culture of intestinal explants allows preservation of the normal histological structure *in vitro* [20]. The pig jejunal explant model has previously been used to study the digestive effects of the mycotoxin DON [21,22,23], and to analyze the toxicity of mixtures of mycotoxins [24]. The present work was designed to compare the acute impacts of DON and NIV on pig jejunal mucosa. The above two models were used in parallel. First, a dose-response study with explants was carried out to estimate the toxic dose for DON and NIV on the mucosa, then, jejunal loops were injected with the two toxins at the selected toxic dose. In the two models, the results, assessed after 4-h of exposure, were concordant, showing a greater impact of NIV compared to DON on the intestinal mucosa.

## 2. Results 

### 2.1. Explants Model 

#### 2.1.1. Histological Analysis before and after Incubation and Effect of DMSO

**Figure 1:**
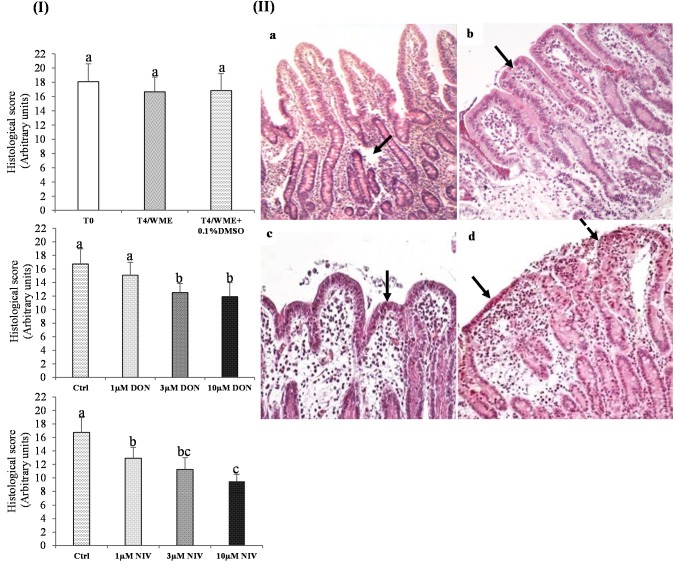
Histological scores of jejunal explants. (**I**) Explants exposed to different treatments: T0 (Time 0H, before culture ), T4/WME (4 h in William’s medium E), T4/WME + 0.1%DMSO (dimethylsulfoxyde), DON (deoxynivalenol) or NIV (nivalenol): 1, 3 and 10 µM. Values are mean ± SEM. (**II**) Effect of DON and NIV on the histological score after 4 hours of exposure. Values are mean ± SEM. a, b, c scripts are different at *p ≤* 0.05 by Tukey’s test (**II**) (**a**) Jejunal explant uncultured (T0; *n =* 12). Slight dilatation of the lymphatic vessels (arrow), HE (hematoxylin-eosin), ×200; (**b**) explants exposed to WME with 0.1% DMSO (DMSO *n =* 42). Edema of the *lamina propria* and mild villus atrophy (arrow), HE ×200; (**c**) 3 µM DON-exposed explant. Moderate fusion and cubic epithelial cells (arrow) (HE, ×200); (**d**) 10 µM NIV-exposed explant. Fusion and atrophy of villi with severely flattened epitelium (arrow) and apical denudation of villi (dotted arrow) (HE, ×200).

First, the effects of the culture and of DMSO on the histology of the jejunal explants were investigated. The explants were observed microscopically and scored from 22 (no lesion) to 0. Before incubation (T0), the scores were between 16 and 21 for all explants (Figure 1 panel I). The histological lesions observed were mild edema in the *lamina propria* and slight dilatation of the lymphatic vessels, resulting in an average score of 18 ± 2 (Figure 1 panels I and IIa).

After incubation in Williams E Medium (WME) for 4 h, with or without DMSO, the scores did not differ significantly from those of the non-incubated explants (Figure 1I), although flattened villi were apparent after this incubation period (Figure 1IIb). The mean villus height was 141 ± 29 µm in the explants incubated with WME alone and 147 ± 41 µm in the explants incubated with DMSO and did not differ significantly from the T0 results. No statistically significant difference was observed between the different incubation groups, with 0.1% DMSO, or without DMSO (Figure 1I). The scores of control explants with or without DMSO were grouped into a single 4-h culture control group for subsequent analyses (*n =* 84).

#### 2.1.2. Effect of Mycotoxins on the Histological Scores

Each treatment, DON (1–10 µM) and NIV (1–10 µM) induced a dose-dependent decrease in the histological scores of the jejunal explants after 4 h of exposure (*p <* 0.01) (Figure 1II). In the explants exposed to DON, the main morphological change was coalescence with moderate fusion of villi. Lesions included cubic epithelial cells instead of the cylindrical epithelial cells seen in the control, areas of edema in the *lamina propria*, villus atrophy and apical denudation of villi with focal loss of apical enterocytes (Figure 1IIc). In the group treated with NIV, the changes were similar to those of the group exposed to DON but both the flattening of the epithelial cells and apical denudation of the villi were more severe (Figure 1IId). The individual treatments with the mycotoxins DON and NIV resulted in a significant decrease of the histological score from doses of 3 µM and 1 µM, respectively. The corresponding scores were reduced to about 70% of the control explants by 3 µM and 10 µM DON or 1 µM NIV, to almost half the mean score of control explants (59% ± 6%) by the highest NIV concentration (Figure 1II). So, NIV showed greater toxicity than DON in the explant model, with a lowest observed effect concentration of 1 µM.

**Figure 2:**
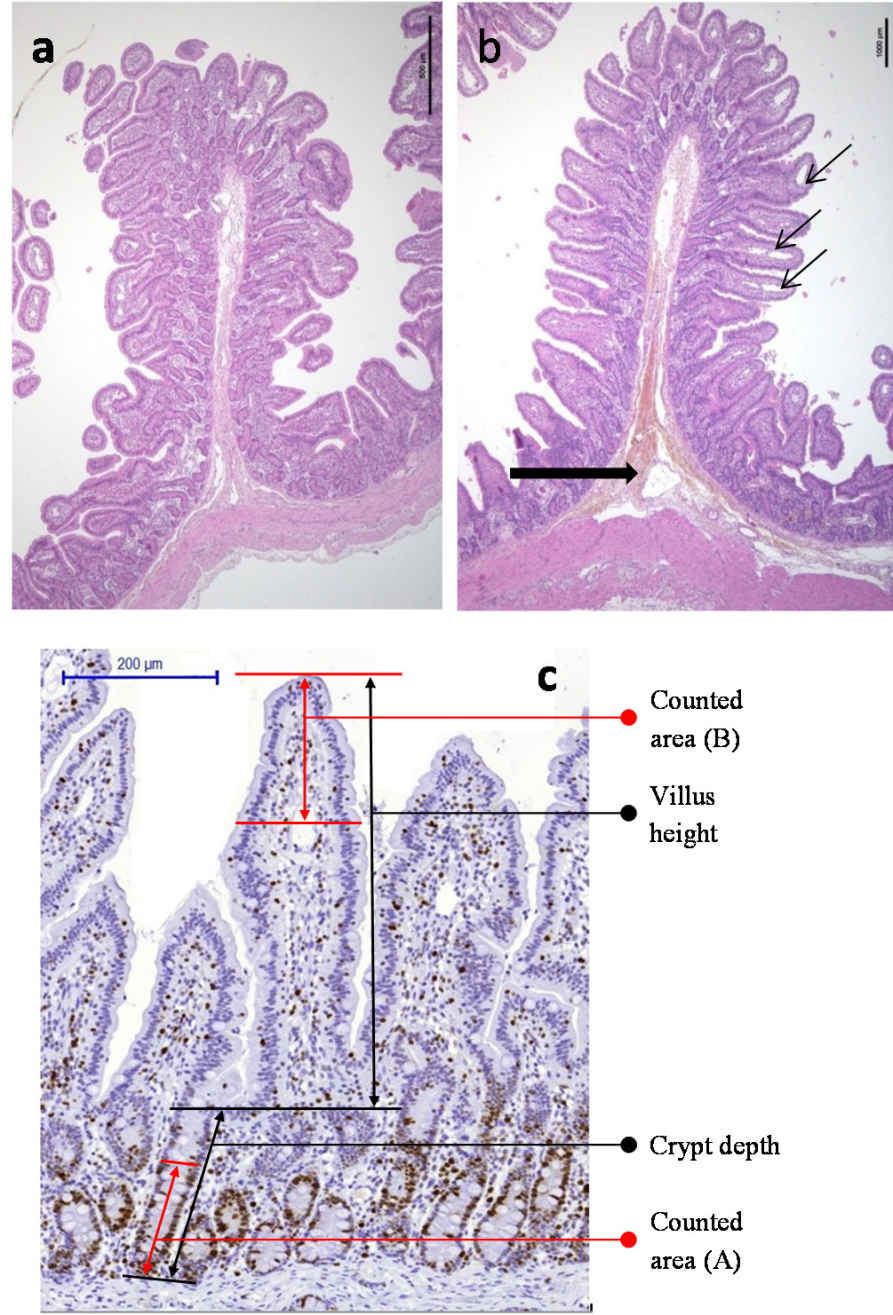
Jejunum morphology in a non-loop segment (**a**) and in a control loop (**b**) showing vascular changes in the submucosa (large arrow) and edema of the villi central lymphatic vessels (thin arrows); (**c**) Ki-67 immunostaining in a control loop, showing the methodology for morphometric and proliferation assessments.

### 2.2. Loops Model

#### 2.2.1. Comparison of Loops Segments with Non-Loops Segments

The surgery induced vascular disorders in the loops, more pronounced in the serosa and submucosa layers, with moderate interstitial edema, congestion, focal blood extravasation and moderate focal dilation of the central lacteal in the villi tips (Figure 2b). The proliferation and apoptosis counts did not differ between the control loops and non-loops segments, and were subsequently used as endpoints after toxins exposure.

#### 2.2.2. Effect of DON and NIV Exposure on Morphometry in the Loops

**Figure 3:**
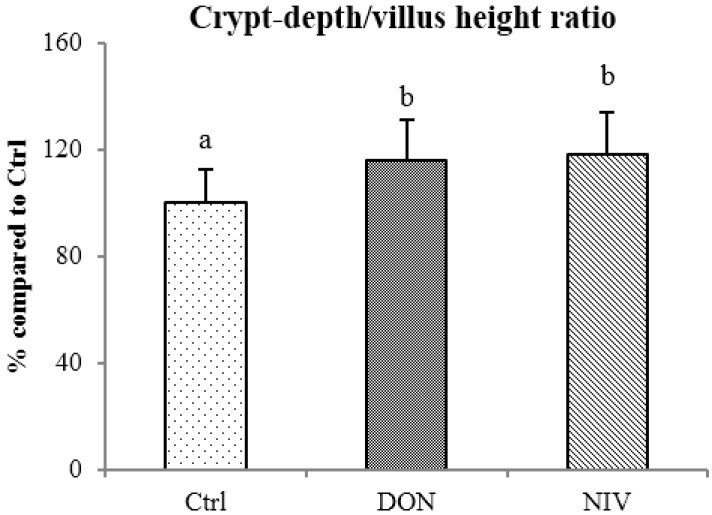
Morphometric analysis of the jejunum loops: crypt-depth to villus-height ratio after DON and NIV exposure at 10 µM for 4 h. Mean values ± SEM expressed as % of the control group; a, b scripts are different at *p ≤* 0.05; Tukey’s test; *n =* 3 to 6 loops, 30 well-oriented villi and crypts per loop.

The mean crypt-depth to villus-height ratios after DON (0.99 ± 0.15) and NIV (1.01 ± 0.16) treatments were increased by 15%–20% compared to the control ratio (0.86 ± 0.11) without significant difference between DON and NIV (Figure 3).

#### 2.2.3. Effect of DON and NIV Exposure on Proliferation in the Loops

**Figure 4:**
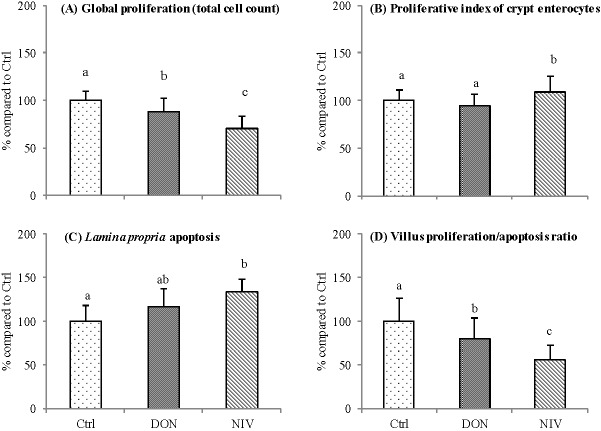
Proliferation and apoptosis in the jejunum loops after DON and NIV exposure at 10 µM for 4 h. Mean values ± SEM expressed as % of the control group (Ctrl) (**A**) total cell proliferation at villus tip (upper one-third), *p <* 0.001; (**B**) proliferative index of crypt enterocytes, *p =* 0.001; (**C**) *lamina propria* apoptosis at villus tip (upper one-third), *p <* 0.001; and (**D**) total proliferating cells to total apoptotic cell ratio, at villus tip *p* < 0.001. a, b, c scripts are different at *p* *≤* 0.05; Tukey’s test; *n =* 3 to 6 loops, 20 villi/loop.

At the villus tip, a significant decrease was observed in the total cells proliferation compared with control loops. NIV exposed loops showed a significant 30% decrease in the number of cells proliferating in the mucosa (*p <* 0.001), while DON-exposed loops showed a 13% decrease compared with the controls (Figure 4A). At the crypt level, proliferative index of crypt enterocytes was increased only after NIV treatment (*p =* 0.001, Figure 4B).

#### 2.2.4. Effect of DON and NIV Exposure on Apoptosis in the Loops

At the villus tip, the number of total apoptotic cells was higher in NIV-exposed loops than in the controls. A tendency was detected for the enterocyte apoptotic index (*p =* 0.057) with mean values of 2.36% ± 1.12%, 2.57% ± 1.52% and 3.43% ± 2.55% for the control, DON and NIV, respectively. The total-cell proliferation to total-cell apoptosis ratios at villus tip showed a significant decrease (*p <* 0.001) with values of 6.98 ± 1.84, 5.60 ± 1.65 and 3.89 ± 1.2 for the controls, DON and NIV, respectively. NIV reduced the total-cell proliferation to total-cell apoptosis ratio at the tip of the villus by 44% compared to the controls, while DON reduced this ratio by only 20% (Figure 4D).

In *lamina propria,* the number of apoptotic cells was significantly increased by NIV (*p <* 0.001, Figure 4C).

## 3. Discussion

Two alternative models were used in this study to analyze intestinal mucosal toxicity, a major target for xenobiotics. An explant model, previously shown to be sensitive [23,24], was first used to demonstrate dose-related toxicity following *in vitro* exposure to DON and NIV, and a higher toxicity of NIV. This result was then confirmed *in vivo*, using the intestinal loops model.

### 3.1. The Jejunum Explants and Loops Alternative Models Reduce the Number of Animals

These two alternative models enabled to reduce the number of animals in experiments, according to the 3Rs recommendations, as the explants or loops are the experimental unit and not the whole animal. Pig intestinal explant culture represent a relevant model for investigating the effects of feed and food contaminants, due to the relevance of the pig model-species in relation to humans, and its high sensitivity to mycotoxins. In the current study, before analyzing the effects of the mycotoxins DON and NIV on the scores, the histological scoring was refined and demonstrated the absence of impact of DMSO up to 0.1% as solvent. This study illustrates for the first time, to the best of our knowledge, the use of intestinal loops in toxicology. However, the scoring system developed for the explants was not suitable to use in the loops model, because of the inter-loops variations brought about by the surgery-induced vascular changes (edema in the *lamina propria*). Proliferation and apoptosis were used to assess the *in situ* mucosal changes, being quantifiable biomarkers specifically affected *in vitro* and *in vivo* by the trichothecenes [14,25,26,27,28].

### 3.2. Acute Exposure to NIV More Toxic in Vitro on Jejunum Mucosa than DON

Our study revealed higher intestinal mucosa changes after acute exposure to NIV than to DON exposure, both *in vitro* and *in vivo*. Following *in vitro* 4-h single exposure, both trichothecenes induced a dose-related decrease of the explants scores, NIV showing a higher toxicity than DON. Significant changes were observed in explants exposed to 3 and 10 µM DON. The main histological changes were focal enterocyte desquamation, moderate atrophy and fusion of villi, in accordance with previous studies [21,23,27]. The 30% reduction of the histological score following 10 µM DON was similar to the results obtained by Basso *et al.* [21] who reported a 37% decrease of the histological score. In our experiment, all doses of NIV significantly affected the histological scores of the explants. NIV toxicity on intestinal cells *in vitro* has already been reported. For example, NIV decreased dose-dependently the viability of Caco-2 and IPEC-J2 cells [29,30]. In a previous study, the reduction of IEC-6 viability due to treatment with NIV was related to apoptosis induction [26]. The less severe toxic effect of DON, as compared to NIV, on intestinal explants is also in accordance with previous studies, which demonstrated that NIV exerted a stronger effect than DON on both intestinal and non-intestinal cell lines [26,28,29,31,32].

### 3.3. Acute Exposure to NIV More Toxic in Vivo on Jejunum Mucosa than DON

In the loops model, as in the explants study, the intestinal toxicity of NIV was higher than that of DON. At 10 µM, both DON and NIV increased the crypt-depth to villus-height ratios, reflecting intestinal damage *in vivo,* as described in conventional animal experiments [33]. At 10 µM, DON and NIV induced *in vivo* *lamina propria* apoptosis and decreased total cells proliferation at the villus tip in the loops model, while only NIV increased the enterocytes proliferation index at the crypt level. So, our study shows that the *lamina propria* cell populations are the most sensitive target of the jejunum mucosa, after DON and NIV exposure. The major cell populations of *lamina propria*, besides connective tissue cells, are immune cells, with high proliferative rate, recognized as the most sensitive cells to DON toxicity. These targets have been previously described to explain the modulation of the intestinal immune response induced by DON and other trichothecenes [34]. Few studies have analyzed the action of NIV on intestinal morphology. Chronic ingestion of NIV induced gastrointestinal erosions in young pigs (2.5 or 5 mg/kg) [15], whereas C57BL/6 mice exposed to NIV subchronically or chronically by feeding did not show any alterations in the histological architecture of small intestine [35,36]. These differences could be related to the highest sensitivity of the pig, reported to be the most sensitive species to mycotoxins [6,37]. In the present study, the proliferation mucosal response at crypt level was significant for NIV. These results are in accordance with previous comparative studies of the two toxins, *in vitro* or *in vivo*, with other endpoints than the intestinal target. For example, NIV showed higher anorectic potency in mice [38].

### 3.4. DON and NIV Induced Apoptosis in Vivo on Loops

The intestinal effects observed after 4-h exposure to DON and NIV can be mediated by oxidative stress, inducing intestinal cell membrane alteration and apoptosis. DON-induced oxydative stress has been shown in splenic tissue in a rodent model [39], as well as lipid peroxidation *in vitro* in HepG2 cells exposed to levels similar to the present study [40]. The alterations caused by DON in jejunal explants have been correlated to MAPKs signaling pathway activation [22], and to up-regulation of pro-inflammatory cytokines [41].

### 3.5. Relevance of the Results for Risk Characterization

The effects of mycotoxins were assessed at realistic concentrations in the present study, considering the concentrations of mycotoxins to which the consumer can be exposed via food. The results are therefore of high relevance for risk characterization of DON and NIV exposure. DON concentrations of 0.16–2 μg/mL (0.5–7 μM) in the human gut can be considered as realistic [42]. The lower concentration corresponds to the mean estimated daily intake of French adult consumers on a chronic basis [43]. The higher concentration is the simulated levels that can be attained after the consumption of heavily contaminated food, and is occasionally encountered. The amount of NIV in cereal products varies considerably between different countries across the world (from 20–60 µg/kg in France, to 584–1780 µg/kg in China) [44]. Significant architectural and lesional alterations were observed from doses of 1 µM NIV in this study, which is consistent with the levels plausibly encountered in the gastrointestinal tract after the consumption of heavily contaminated food.

## 4. Materials and Methods 

### 4.1. Animals

For explants sampling, six 4–5 week-old crossbred piglets were used, housed in the animal facility of the INRA ToxAlim Laboratory (Toulouse, France). For the loops experiment, three two-month-old Large White female pigs were used and housed in the animal facility at INRA Nouzilly. All animals were fasted for 6 h before explants sampling or loops surgery. The experimental procedures were conducted in accordance with European Guidelines for the Care and Use of Animals for Research Purposes and were approved by the INRA local ethical committees for animal experimentation (C2EA-86 for explants, and “*Comité d’Ethique en Expérimentation Animale Val de Loire*”, C2EA-06, for the loops experiments).

### 4.2. Toxins 

DON was acquired from Sigma (St Quentin Fallavier, France) and NIV from Waco Pure Chemical Industries LTD (Osaka, Japan). Stock solutions of these mycotoxins were dissolved in dimethyl sulfoxide (DMSO Sigma, Saint-Quentin Fallavier, France) at the following concentrations: 15 mM DON and 10 mM NIV for explants, and at 30 mM DON and NIV for the loops experiments. These stock solutions were stored at −20 °C. Working dilutions were prepared in William’s medium E (WME-Sigma, Saint-Quentin Fallavier, France) for the explants, and in physiological saline solution for the loops. The concentrations range for the dose-response explants study was 0–10 µM, selected after preliminary explants cultures with 0.1 to 30 µM of each toxin.

### 4.3. Jejunum Explants Experiment (in Vitro)

#### 4.3.1. Jejunal Explants 

The procedure for the culture of explants was as previously described [23,24]. Explants were incubated for 4 h with WME at 37 °C under a CO_2_-controlled atmosphere with orbital shaking. Uncultured control tissue was placed in fixative immediately after dissection, as time 0 controls (T0, *n =* 12, two explants/pig). In view of the possible effects of DMSO on intestinal morphology, the final concentration of 0.1% DMSO corresponding to the highest DMSO concentration in the working dilutions was tested in 42 explants (with and without DMSO: 84 explants). Twelve explants were exposed to purified DON and NIV at each of the concentration 1, 3 and 10 µM, for 4 h, respectively (two explants/pig for each condition).

#### 4.3.2. Histological Scoring 

For histological analysis, the explants fixed in 10% formalin (VWR, Strasbourg, France) were embedded in paraffin (VWR) and sectioned at 3–5 µm thickness parallel to the villus axis and stained with hematoxylin (VWR) and eosin (CML, Nemours, France) (HE) using standard procedures. The resulting slides were analyzed independently by two observers, at 100× magnification.

The histological changes were evaluated using a tissue scoring system [23] with minor modifications. The scoring system included both architectural and lesional criteria, as shown in Table 1. The maximum score was attributed to the T0 tissue, before incubation, for each criterion. The architectural score included the number of villi per explant and the fusion of villi. This latter was expressed as the 97.5th percentile of the percentage of fused villi (number of fused villi/non fused villi 100×). The score of 3 for villus fusion corresponded to a maximum of 11% fused villi. At least 25 villi needed to be counted per explant to obtain the score of 3.

The lesional score included morphology of enterocytes (score 3 for columnar epithelium), the degrees of edema and apoptosis in the *lamina propria* (score 2 for slight flattening of villi), and the extent of discontinued epithelium qualified as apical denudation of villi. This endpoint was quantified by the 97.5th percentile of the percentage of the villi showing apical denudation (score 3 for T0 explants). For explants lesions, score 3 corresponded to a maximum of 10% apical denudation and scores of 2, 1, and 0, to 11%–40%, 41%–70%, and 71%–100%, respectively. For lesion of the *lamina propria*, localized edema was scored as 1, whereas multifocal edema and apoptosis were scored as 0. The total score was calculated by taking into account the degree of severity for the lesions (severity factor). For each lesion, the score (according to intensity or observed frequency) was multiplied by the severity factor of 2. The total score for each explant was then obtained from the sum of each criterion. Each score value was the result of 2 explants/pig/condition. The maximum score (22 points) indicated overall integrity of the intestine. The histological scoring system was applied to compare the microscopic changes observed after 4-h exposure of the explants to DON and NIV.

**Table 1:** Explants histological scoring: endpoints used and severity factor.

Score component	Criteria (severity factor)	End-point	Score
Lesional part of the Score	Enterocytes morphology (2)	Columnar epithelium	3
		<50% cuboid epithelium	2
		>50% cuboid epithelium	1
		Flattened epithelium	0
	Apical denudation of villi (2)	0%–10%	3
		11%–40%	2
		41%–70%	1
		71%–100%	0
	Lesions of *lamina propria* (2)	No lesions, slight flattening of villi	2
		Localized edema and apoptosis	1
		Multifocal edema and apoptosis	0
Architectural part of the Score	Villi fusion (1)	0%–11%	3
		12%–40%	2
		41%–70%	1
		71%–100%	0
	Number of villi (1)	≥25	3
		16–24	2
		5–15	1
		≤4	0

### 4.4. Jejunum Loops Experiment (in Vivo)

#### 4.4.1. Jejunal Loops Injection and Sampling

A 1-m long segment of intestine was surgically prepared in the jejunum, to constitute the loops as previously described [19,45]. This segment was then subdivided into consecutive segments, designated as “loops” (10 cm long, 6 loops), separated by “inter-loops”. Three treatments, control (Ctrl), DON and NIV at 10 µM concentration were used for each of the 3 pigs (1 to 2 loops/pig for each condition, *n =* 6 loops for the controls) by injecting 3 mL of each test condition into each loop. Four hours after surgery, the pigs were euthanized by barbiturate overdose (pentobarbital, Vetoquinol, Lure, France) and the created jejunum-loop segments were collected. These loops were washed twice with physiological serum prior to fixation. In addition, a non-loop segment was sampled and processed in parallel.

#### 4.4.2. Histological Processing

A routine histological processing sequence (from 10% buffered formalin to paraffin block) was used. Paraffin sections 4-µm thick were stained with HE to assess architectural changes and immunohistochemically labeled (IHC) to assess proliferation and apoptosis.

#### 4.4.3. Immunohistochemistry

Two commercial antibodies were used as previously described [46,47]. Briefly, four-micrometer paraffin-embedded transverse sections from formalin-fixed jejunum specimens were dewaxed in toluene and rehydrated by an acetone bath then deionized water. Antigen retrieval was performed in 10 mM citrate buffer pH 6.0 for 30 min in a water bath at 95 °C. Cooled sections were then incubated in Dako peroxidase blocking solution (Dako S2023) to quench endogenous peroxidase activity. Non-specific binding was blocked by incubation in normal goat serum (dilution 1:10, Dako X0902) for 20 min at room temperature. The primary antibodies were anti-Ki-67antigen (Dako M7240, dilution 1:50) and anti-active caspase-3 (R&D system, AF835, dilution 1:300). Sections were incubated with primary antibodies for 50 min at room temperature (RT). Bound primary antibodies were detected with EnVision™ + Horse Radish Peroxydase (HRP) Systems (Dako, K4061) 30 min at RT. Peroxidase activity was revealed by 3,3′-diaminobenzidine tetrahydrochloride substrate (Dako K3468). Finally, sections were counterstained with Harris hematoxylin, dehydrated and coverslipped.

#### 4.4.4. Architectural Changes

Eclipse E400 Nikon microscope, with DS-FI camera driven by NIS-D element software (Nikon) was used to capture images (100× magnification) and take the measurements for architectural evaluation of the digestive mucosa. A total of about 30 well-oriented villi and crypts per loop were selected on each section. A villus was measured from the tip to the shoulder (crypt-villus junction) and a crypt was measured from the shoulder to its base (Figure 1c). The crypt-depth to villus-height ratio was calculated to assess the intestinal architectural changes.

#### 4.4.5. Proliferative and Apoptosis Indexes

Proliferative and apoptotic cells were counted after immunohistochemistry in several sites on the mucosa. A minimum of 20 well-oriented villi and crypt units were assessed on scanned marked slides (Panoramic 250 Flash II–3D Histech), and analyzed with Pannoramic Viewer software (v. 1.15.2, 3DHISTECH Ltd, Budapest, Hungary,). Proliferative cells were counted in the upper one-third of the villi, *i.e.* villus tip (positive cells/total cells: *lamina propria* plus epithelial cells), and in the bottom two-thirds of the crypts from the basis (proliferative index of crypt enterocytes) (Figure 1c). For apoptosis, the total cells counts, *lamina propria* counts and villus enterocytes counts were measured in villus tip (upper one-third). The enterocyte apoptotic index was calculated by dividing the positive enterocyte number by the total number of enterocytes (×100) in the villus tips while the proliferative index of crypt enterocytes was calculated by dividing the number of positive enterocytes by the total number of enterocytes (×100) in the crypt bases (Table 2).

**Table 2:** Summary of the cell counts and indexes used for assessing proliferation and apoptosis in loops.

Endpoint	Counted area (Figure 2)	Cells counts	Indexes
*Proliferation*			
	Villus tip	Total cells: *lamina propria* cells + enterocytes	
	Crypt bases	Crypt enterocytes	Proliferative index of crypt enterocytes: number of positive enterocytes/total number of enterocytes (×100)
*Apoptosis*			
	Villus tip	Enterocytes *Lamina propria* cells (mainly immune cells) Total cells: *lamina propria* + enterocytes	Enterocyte apoptotic index: number of positive enterocytes/total number of enterocytes (×100)
*Ratio Proliferation/Apoptosis*			
	Villus tip	Total cells: *lamina propria* cells + enterocytes	Total-cell proliferation to total cell apoptosis ratio

### 4.5. Statistical Analysis

The two experiments were designed as randomized blocks. The explants and loops data are presented as means ± SEM, expressed as percentages of the control values. Plots of fits *versus* residuals followed by Bartlett’s test, and normal plots of the residuals by Anderson-Darling’s test were carried out to confirm the assumptions of homogeneity of variances and the normal distribution of residuals, respectively. If these assumptions did not hold, the data were normalized and homogenized by log10 or square root transformation prior to being analyzed. All tests were performed using the MINITAB package software (V.13.0, Minitab Inc., State College, PA, USA). The data were analyzed by applying the GLM option of ANOVA analysis, followed by pairwise comparisons, Tukey’s or Bonferroni’s tests. The datasets for the control loops and non-loops were analyzed with the Wilcoxon matched pairs test and paired t-test for apoptosis and proliferation counts, respectively [48].

## 5. Conclusions

To conclude, the present study shows that pig intestinal explants and loops provide concordant results and permit investigating the digestive effects of DON and NIV with a reduced number of animals (implementation of the 3Rs). Acute NIV exposure induced mucosal changes at a lower concentration than DON *in vitro*. *In vivo*, *lamina propria* cells showed a higher sensitivity than enterocytes to NIV-induced apoptosis. Our results demonstrate that NIV toxicity is not similar to DON on the digestive target, highlighting the need of a specific hazard characterization for NIV health risk assessment.
